# Detection of High-Grade Cervical Intraepithelial Neoplasia by Electrical Impedance Spectroscopy in Women Diagnosed with Low-Grade Cervical Intraepithelial Neoplasia in Cytology

**DOI:** 10.3390/life13112139

**Published:** 2023-10-31

**Authors:** Georgios Panagakis, Ioannis K. Papapanagiotou, Charalampos Theofanakis, Paraskevi Tsetsa, Adamantia Kontogeorgi, Nikolaos Thomakos, Alexandros Rodolakis, Dimitrios Haidopoulos

**Affiliations:** 1 Department of Obstetrics and Gynecology, Alexandra General Hospital, University of Athens, 4-2 Lourou, 115 28 Athens, Greece; gpssas@yahoo.he (G.P.); gpapamd@hotmail.com (I.K.P.); charalampostheofanakis@yahoo.com (C.T.); bibitse@hotmail.com (P.T.); thomakir@hotmail.com (N.T.); a.rodolaki@gmail.com (A.R.); dimitrioshaidopoulos@gmail.com (D.H.); 2 Department of Obstetrics and Gynecology, Attikon General Hospital, University of Athens, Rimini 1, 124 62 Haidari, Greece

**Keywords:** cervical intraepithelial neoplasia, colposcopy, electrical impedance spectroscropy, high-grade CIN zedscan device, low-grade CIN

## Abstract

The authors attempt to address the importance of timely detection and management of cervical intraepithelial neoplasia (CIN) to prevent cervical cancer. The study focused on the potential of electrical impedance spectroscopy (EIS) as an adjunct to colposcopy, aiming to enhance the accuracy of identifying high-grade cervical lesions. Colposcopy, a widely used technique, exhibited variable sensitivity in detecting high-grade lesions, which relies on the expertise of the operator. The study’s primary objective is to evaluate the effectiveness of combining colposcopy with EIS in detecting high-grade cervical lesions among patients initially diagnosed with low-grade CIN based on cytology. We employed a cross-sectional observational design, recruiting 101 women with abnormal cervical cytology results. The participants underwent colposcopy with acetic acid and subsequent EIS using the ZedScan device. The ZedScan results are categorized into color-coded probability levels, with red indicating the highest likelihood of high-grade squamous intraepithelial lesions (HSIL) occurrence. Results revealed that ZedScan exhibits a sensitivity rate of 89.5% and a specificity rate of 84% for detecting high-grade lesions. Colposcopy, on the other hand, recorded a sensitivity rate of 85.5% and a specificity rate of 92%. The agreement rate between ZedScan and biopsy is 79.2%, as indicated by a kappa coefficient of 0.71, while the agreement rate between colposcopy and biopsy is 74.3%, with a kappa coefficient of 0.71.

## 1. Introduction

Cervical cancer is one of the top four most common malignant tumors for female patients and one of the leading causes of death from cancer among women. The World Health Organization reported more than half a million new cases in 2020 and a high cancer-related mortality rate (342,000 deaths) [1,2]. The essential cause of cervical cancer has been identified as persistent infection with high-risk human papillomavirus (HPV) [3,4]. Thus, detecting and treating the precursor lesion, cervical intraepithelial lesion (CIN) is essential in order for any screening program to succeed and can prevent the onset of cervical cancer.

The traditional diagnostic procedure known as colposcopy is carried out after a negative cervical cancer screening test. It involves magnifying the view of the cervix up to 30 times with a colposcope. The squamocolumnar junction (SCJ) and the transition zone are primarily the focus of the study. The dynamic area known as the transformation zone is where glandular cells eventually give way to squamous cells through metaplastic processes, increasing the risk of neoplasia [5].

The cervix is examined during colposcopy after a 35% acetic acid solution is applied during visualization of the cervix with acetic acid (VIA). The acidic solution dehydrates cells after about 30 to 90 s, causing squamous cells with relatively big or dense nuclei to reflect light and look white [6]. These are known as “acetowhite changes.” Furthermore, against this white background, abnormal blood vessels and vascular patterns become clearer and more visible. Similarly, Lugol’s iodine can be administered to the cervix, making dysplastic lesions easier to identify. Lugol’s iodine is a substance that becomes brown or black when it comes into contact with glycogen, which is found in adult squamous epithelium. Because of weak cellular differentiation, precancerous lesions and cancer contain little or no glycogen and will turn various hues of yellow following the application of Lugol [7].

Colposcopy is widely used as part of screening programs to detect and treat such lesions, mainly high-grade cervical intraepithelial grade 2+ lesions (HG-CIN, CIN 2+) and cervical cancer. Its sensitivity for detecting high-grade lesions ranges from 55.9% to 60% [8,9,10,11] and can reach 85.6% if the number of retrieved biopsies is increased or endocervical curettage is added [12,13,14]. Sauvaget et al. [15] conducted the most complete meta-analysis in which colposcopy was utilized as a primary screening modality. According to the authors, the overall sensitivity is 80%, the specificity is 92%, the negative predictive value is 99% and the positive predictive value is 10% [15]. Furthermore, they concluded that region, screening provider training level, setting, and size of the study population had no effect on colposcopy accuracy. The substantial negative predictive value reported by Sauvaget et al. [15] was replicated by Sankaranarayanan et al. [16] in an Indian longitudinal study. Only 25 of the 23,000 VIA-negative women examined in this trial had cervical cancer within the subsequent eight years. This means that women who have had a colposcopy and had a negative screening result are unlikely to suffer from cervical cancer in the near future [16]. 

However, effectiveness depends on the experience and training of the colposcopist, the prevalence of the disease in the study population, and the number of biopsies performed [17]. The inadequate evaluation of a colposcopic image can result either in failure to detect disease or unnecessary treatment in the absence of the disease. Electrical impedance spectroscopy (EIS), as a real-time adjunct to colposcopy, has been reported to improve the performance of colposcopy [17,18,19]. In fact, studies have demonstrated that EIS has been frequently employed in melanoma patients to differentiate between normal and abnormal skin lesions based on their modified cellular structure as a result of transformation into cancer [20,21]. EIS, according to researchers, could be used in gynecology and constitutes a promising and quick approach for detecting aberrant cellular alterations in cervical epithelium [14,22,23,24]. Apart from the loss of stratification and differentiation and higher nuclear-cytoplasmic ratio, it appears that CIN development can cause an up to six-fold increase in extracellular space, which decreases impedance at low frequencies in CIN. As a result, the original squamous epithelium has a high impedance, but high-grade CIN has a lower impedance [14,24,25,26].

The primary endpoint of our study was to evaluate the effectiveness of the combination of colposcopy and EIS in detecting high-grade intraepithelial cervical lesions in patients referred to colposcopy due to an initial diagnosis of a low-grade lesion on cytology. Therefore, our ultimate goal was to identify patients underdiagnosed or patients not indicated to undergo further evaluation, thus, reducing morbidity, economic cost, and psychological stress. Based on the above, the purpose of the study is to test the research question whether the additional examination with zedscan in the colposcopy routine increases sensitivity in the detection of LGSIL in women with LGSIL.

## 2. Materials and Methods

### 2.1. Design

This study was conducted at the Colposcopy Unit of «Alexandra» General University Hospital in real-world settings and could be characterized as a real-world study due to the sample size. All eligible women were initially referred to colposcopies to our institution due to abnormal cytology results and, subsequently, electrical impedance measurements were received from each woman with a specific measuring device.

To evaluate the diagnostic method, an analysis was performed at 3 levels:

1. A colposcopy examination, with biopsies taken from suspicious cervical lesions;
2. ZedScan spectroscopy, with extra biopsies of areas that had been identified as high risk for HGSIL;
3. Histopathological analysis of tissues with suspected damage.

**Figure 1:**
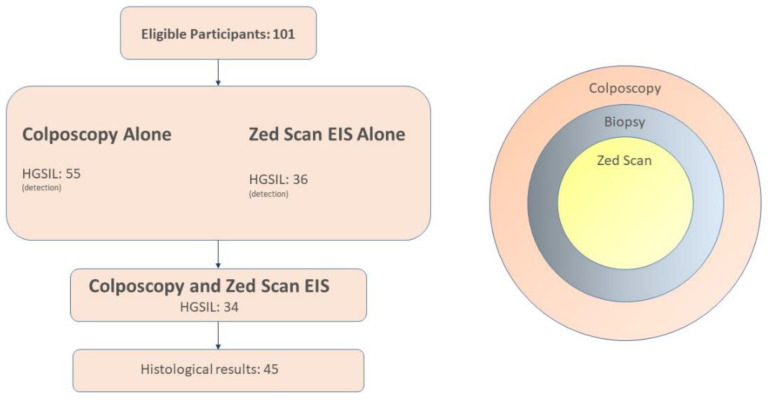
A schema outlining the thought process behind the methodology followed for the design of this study.

Furthermore, based on the international literature and to ensure the participants’ safety, we thought it was reasonable, when colposcopy was negative and a high-grade disease was found only by a ZedScan-directed biopsy, to be also regarded as an increase in detection. A patient was also considered negative for HSIL if the colposcopy was normal, there were no visible lesions, the ZedScan test was negative, and the patient had been referred with low-grade cytology (ASCUS, LSIL). To summarize, the patients served as their controls, having undergone both procedures during the same examination, enabling a comparison of the rate of detection for high-grade lesions for colposcopy alone versus colposcopy in combination with ZedScan (Figure 1). 

### 2.2. Participants

**Figure 2:**
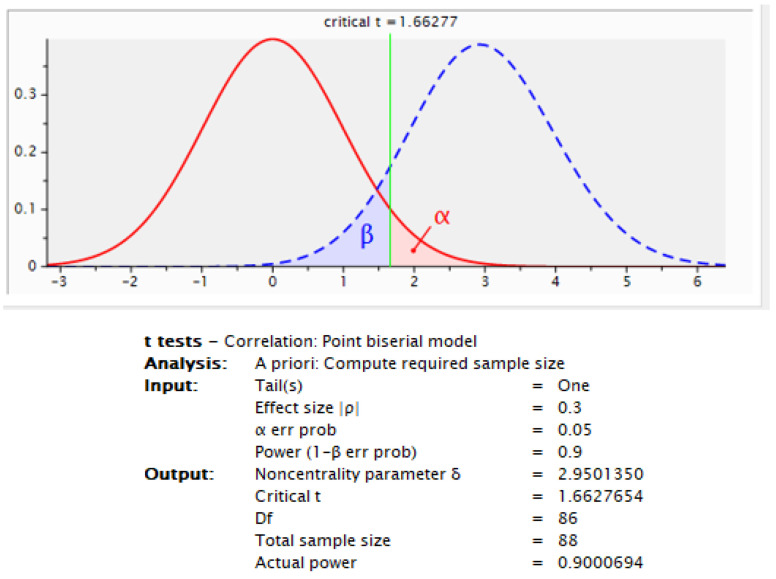
G Power analysis chart.

In total, 101 women were recruited to participate in the study from 2019 to 2022. The sample size for this study was calculated using G Power 3.1 software. Given the effect size, desired power level, and significance threshold, the analysis determined the minimum required sample size for the single-group design. This approach ensured adequate statistical power to detect meaningful effects within the study population. Based on the effect size of 0.3, a desired power of 0.9, and a significance level of 0.05, the software calculated a minimum required sample size of 88 participants to detect significant differences. To account for potential dropouts, we increased the target sample size to 101. This ensured adequate statistical power for the analysis of the primary outcome measures (Figure 2).

### 2.3. Inclusion–Exclusion Criteria

Inclusion criteria: abnormal cervical cytology, LSIL, ASCUS, or cervical inflammatory changes.

Exclusion criteria: pregnancy, vaginal bleeding or active menstruation, cervical cancer, use of vaginal contraceptives or vaginal medication, or refusal to participate in the study.

### 2.4. Intervention

The same diagnostic approach was applied to all women and all diagnostic maneuvers were performed by two senior gynecologists. Colposcopies using Videocolposcope HD-1000 with IRIS software v22.3.0.621 (Medicom, Wroclaw, Poland) were conducted according to established protocols of our hospital colposcopy unit. 

Initially, all women underwent a standard colposcopy with acetic acid for an overview of the cervix and visualization of the possible epithelial lesions. Next, EIS was performed with ZedScan (Zilico Limited, Manchester, UK).

**Figure 3:**
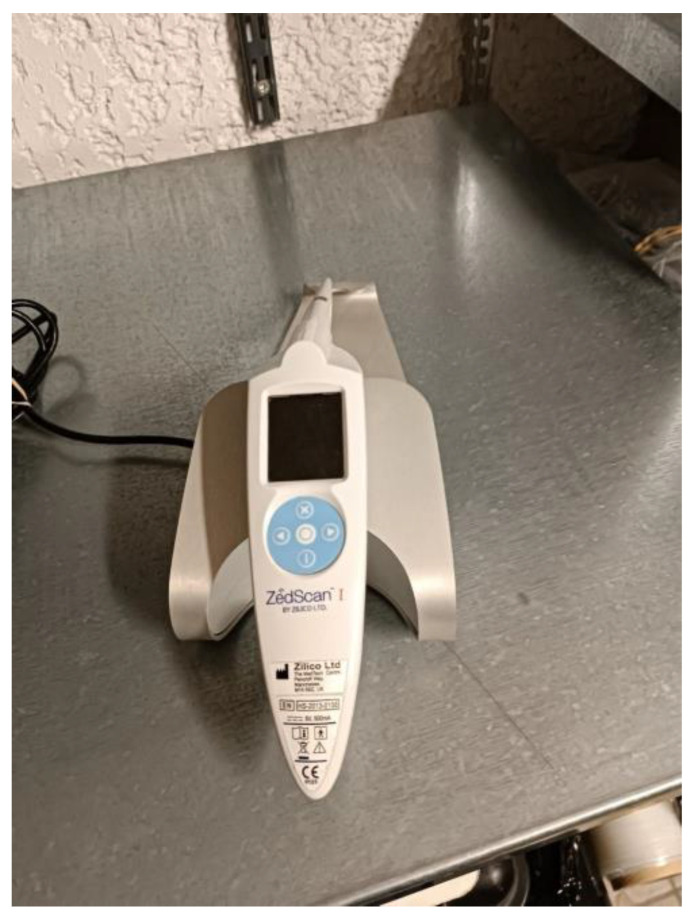
Zed scan handheld unit on base station by ZILICO Ltd. with serial number 3238544605EBGG31 manufactured on 10/2013.

The device ZedScan consisted of a handheld unit with a single-use sensor on the tip of the unit and an integrated screen in the area of the handle (Figure 3). The whole cervical transformation zone (TZ) was observed and 12 measurements were taken from the TZ after 5% acetic acid was applied to the cervix. Measurements were presented and recorded on the handheld unit’s screen.

For the interpretation of the results, 3 colors, red, amber, and green, were used to identify areas with the highest probability of HSIL occurrence. The red color is interpreted as the area with the highest probability for HSIL and the green color is the area with the lowest probability. Intermediate states are depicted in amber and are associated with a low probability of HSIL.

The diagnostic procedure was completed by the application of potassium iodide to the surface of the cervix, identifying abnormalities on the cervical epithelium using the standard method. HSIL was confirmed by diagnostic cervical biopsies or cone biopsies. All biopsy specimens were analyzed by histopathologists with expertise in cervical pathology.

ZedScan’s positivity for CIN was determined based on color changes, with amber or red indicating a positive result, and green representing a negative result. In cases where colposcopy yielded negative results but CIN2+ disease was identified through a biopsy guided by ZedScan, it was categorized as an enhanced CIN2+ detection attributable to ZedScan. Conversely, when both colposcopy and ZedScan were negative, patients were classified as negative for CIN2+. Notably, even in instances of negative results, due to the presence of suspicious cytology (ASCUS, LSIL), biopsies were invariably conducted. The ultimate endpoint for comparison of diagnostic efficacy between colposcopy and ZedScan was the histology reports resulting from these biopsies.

Colposcopy was completed by applying Lugol’s solution to the cervical surface, which assisted in the identification of possible abnormalities without the need for staining. Biopsies were conducted based on colposcopic impressions and/or ZedScan results, and the excised tissues were histologically evaluated at the Hospital’s Pathology Lab.

For cytological assessment, the collected samples were preserved in a PreservCyt/ThinPrep solution (Cytyc Corporation, Marlborough, MA, USA, 1987). The Pap test employed Liquid-Based Cytology (LBC) technology and was processed using the Thin Prep 2000 Processor (Cytyc Corporation, Marlborough, MA, USA, 1987), as per the manufacturer’s guidelines. Thin-layer slides were Pap stained and thoroughly evaluated by a team of professional cytologists specialized in cervical pathology by the standards established in the Bethesda System for Reporting Cervical Cytology, third edition, 2015. Cytological findings were classified as (a) low-grade squamous intraepithelial lesions (LSIL), (b) atypical squamous cells of undetermined significance (ASCUS), (c) negative for intraepithelial lesion or malignancy (NILM), (d) atypical squamous cells of undetermined significance without excluding high-grade squamous intraepithelial lesions (ASC-H), (e) high-grade squamous intraepithelial lesions (HSIL), or (f) squamous cervical carcinomas (SCC).

The Clinic’s Laboratory of Gynecologic Oncology performed the molecular analyses. 1 mm of each Thin Prep sample was transferred to an Aptima Specimen Transfer Tube and processed using the Panther system (Hologic, Marlborough, MA, USA, 1985). The mRNA Aptima assay (Hologic, Marlborough, Massachusetts, United States of America, 1985) functioned as a qualitative method to detect mRNA from 14 different hr HPVs (HPV-16, 18, 31, 33, 35, 39, 45, 51, 52, 56, 58, 59, 66, and 68), utilizing specific probes targeting viral E6 and E7 mRNAs. An amplification process involving transcription-mediated amplification (TMA) was used to amplify DNA copies, which subsequently served as templates for RNA amplification. Probe hybridization was then employed for detection (measured as relative light units, RLU). Controls were integrated to establish a cut-off level, and outcomes were presented as signal-to-cut-off (S/CO) ratios. The study initially adhered to a recommended S/CO ratio cutoff value of 1.0, where an S/CO above 1.0 was automatically regarded as a positive result by the analyzer’s algorithm. The Aptima HPV assay was meticulously conducted according to the manufacturer’s provided instructions.

### 2.5. Statistical Analysis

The study employed a range of statistical methods to analyze the data appropriately. Qualitative variables were presented as absolute and relative frequencies while quantitative variables were presented as mean values with standard deviations (SDs). Proportions were compared using Chi-square tests. The predictive capabilities of the ZED scan and colposcopy were assessed through Receiver Operating Characteristic (ROC) analysis, with the AUC (area under the curve) serving as a measure of overall performance. Logistic regression models assisted in generating linear predictors and comparing AUC values. In clinical trials, the utilization of AUC (Area Under the Curve) analysis, especially in the context of Receiver Operating Characteristic (ROC), is essential for multiple reasons. It facilitates the assessment of diagnostic tests, aiding in the comparison and selection of effective tools for disease identification. AUC also plays a crucial role in evaluating the predictive power of models and biomarkers, providing insights into treatment efficacy and disease progression. Furthermore, it helps identify and assess risk factors associated with specific health outcomes, contributing to a better understanding of disease occurrence and development. AUC analysis assists in determining optimal cut-off points for diagnostic tests, ensuring accurate clinical decision-making. By quantifying prognostic accuracy, it enables tailored treatment strategies and personalized medicine, ultimately enhancing patient care and outcomes in clinical practice [27]. 

The effectiveness of the ZED scan and colposcopy was gauged using metrics like sensitivity, specificity, and positive and negative predictive values. To evaluate the agreement between the ZED scan, colposcopy, and histology, the Kappa coefficient (K) was utilized. The highest Kappa value attainable was 1, signifying perfect agreement; K values equal to or greater than 0.75 indicated excellent agreement, while values surpassing 0.4 indicated acceptable reliability.

All reported *p*-values were two-tailed, and a result was considered statistically significant if it was ≤0.05. The analyses were performed using the SPSS software for statistical analysis (version 22.0, SPSS Inc., Chicago, IL, USA, 1968).

## 3. Results

### 3.1. Demographics

The study comprised a selected cohort of 101 patients, with an average age of 39.8 years, spanning a range from 20 to 64 years. The average age of starting sexual contact was 17 years and of the patients, 42% had more than three sexual partners. Of the sample of 101 women, 78% reported systematic contact without condom use. Furthermore, 68 of the 101 women are married and 52 report having had only one sexual partner in the last 10 years. Of the patients, 63 have at least one child and 47 have had at least one vaginal delivery.

From the other demographics, it is reported that 38% of the women were obese with a BMI > 25, 9% were underweight and the rest were in the normal range. Sixty-one percent of the sample are smokers and 34% suffer from chronic diseases. Detected comorbidities include Systemic Lupus Erythematosus (SLE), type I and II diabetes, and ulcerative colitis. Of the patients, 45% report a history of fungal vaginitis, and 31% report a pathological culture result of vaginal fluid for which they received antibiotics. It is worth noting that 62% report skipping an annual gynecological examination and 28% of women did not undergo a pap test in the last 10 years. Of the examinees, only 14 have been vaccinated with Gardasil, all before the age of starting sexual contact (Table 1).

### 3.2. Cytological-Colposcopical and Histopathological Findings

All individuals were enrolled based on their referral for colposcopy due to an initial diagnosis of low-grade cervical intraepithelial lesion (LGSIL). The subsequent analysis targeted on the outcomes of three diagnostic methodologies: ZedScan, colposcopy, and biopsy, with the resulting data presented in Table 2.

Within this patient pool, 45 cases exhibited CIN2 during histopathological examination, with high-grade colposcopy results observed in 36 instances. Notably, 55 patients were identified as displaying positive (red) ZedScan results. Importantly, no adverse events linked to the use of ZedScan were reported by any of the patients. The pivotal metrics of sensitivity, specificity, positive predictive value, and negative predictive value were calculated by aligning the outcomes of colposcopy and ZedScan examinations with histopathological findings, thus providing a comprehensive assessment of their diagnostic efficacy (Table 3).

**Figure 4:**
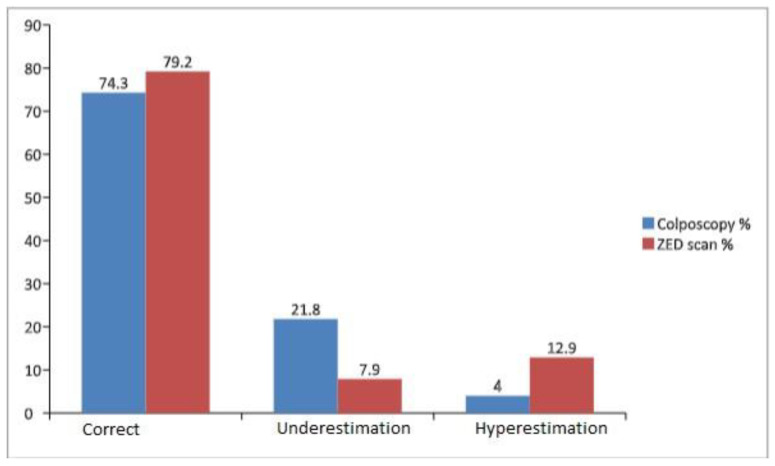
Proportion of overestimated and underestimated cases from ZED scan and colposcopy in association to biopsy results.

Intriguingly, the agreement rate between ZedScan results and biopsy outcomes was notably high at 79.2%, underpinned by a statistically significant kappa coefficient of 0.71 (*p* < 0.001). Similarly, the concordance between colposcopy results and biopsy findings reached an agreement rate of 74.3%, accompanied by a substantial kappa coefficient of 0.71 (*p* < 0.001). Furthermore, the alignment rate between ZedScan and colposcopy findings was 65.3%, marked by a kappa coefficient of 0.58 (*p* < 0.001) (Figure 4).

**Figure 5:**
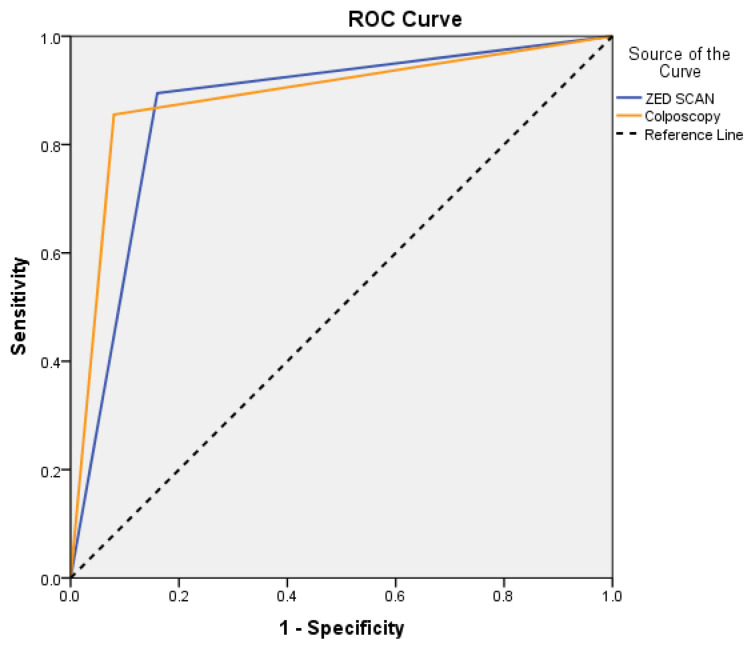
ROC analysis results for the accuracy of ZED scan and colposcopy.

Regarding the diagnostic capabilities of ZedScan, its sensitivity rate stood at 89.5%, indicating its effectiveness in identifying true positive cases. Correspondingly, its specificity rate was calculated at 84%, underscoring its ability to accurately identify true negative cases. Furthermore, the positive prognostic value (PPV) of ZedScan reached 94.4%, while its negative prognostic value (NPV) was 72.4% in predicting biopsy outcomes (Table 4). Notably, the area under the curve (AUC) for ZedScan stood at 0.87 (Figure 5), significantly surpassing the baseline of 0.5 (*p* < 0.001), thereby emphasizing its diagnostic robustness (Table 5).

In parallel, the diagnostic capabilities of colposcopy were revealed, showcasing a sensitivity rate of 85.5% and a specificity rate of 92%. Its positive prognostic value reached 97%, while the negative prognostic value was determined to be 67.6% for predicting biopsy outcomes. The AUC for colposcopy was 0.89 (Figure 5), firmly establishing its diagnostic prowess and superiority over the baseline (*p* < 0.001). Finally, the comparative assessment of ZedScan and colposcopy’s prognostic capabilities yielded intriguing insights. The comparison of AUCs yielded a *p*-value of 0.741, indicating no statistically significant difference between their diagnostic abilities.

Individual statistical analysis was also performed for all risk factors identified in the individual patient recall history. Except for the use of the number of lifetime sexual partners, which was statistically significant (*p* = 0.029 ≤ 0.05), most ZedScan parameters were not statistically significant. Smoking (*p* = 0.069 > 0.05), prophylactics (*p* = 0.134 > 0.05), age (*p* = 0.376 > 0.05), number of children (*p* = 0.765 > 0.05), and HPV vaccination (*p* = 0.067 > 0.05) all had no statistical significance.

Even if statistical crosstabulation illustrates the common distribution between the pairs of variables under consideration, the chi-square test cannot be deemed sufficiently reliable because many cells in the table require low expected frequencies. Given that the values of all variables under consideration are ordinal, we used Spearman’s (rho) nonparametric correlation coefficient, with values close to +1 indicating a strong positive correlation and values close to -1 indicating a strong negative correlation. The number of lifetime sexual partners (*p* = 0.018) is the only statistically significant variable for the two approaches with a substantial positive correlation (*p* = 0.85), which can be verified by the existing bibliography that it corresponds with the emergence of CIN2+. The association can be verified for women who have had more than three partners in their lives. All of the other variables’ correlation coefficients were between 0.4 and 0.7, indicating a moderate correlation, or less than 0.5, indicating a low correlation.

In conclusion, this study analyzed the diagnostic capabilities of ZedScan and colposcopy in a carefully selected patient cohort with low-grade cervical intraepithelial lesions. The thorough evaluation of agreement rates, sensitivity, specificity, positive and negative predictive values, and AUCs collectively delineated the robustness of these diagnostic tools, offering invaluable insights into their potential applications in clinical practice. From the analysis of the demographic characteristics of the sample, only the number of sexual partners is related to the occurrence of CIN 2+, while the rest of the observations follow the data we receive from the international literature.

## 4. Discussion

It is clear that for the early prevention of cervical cancer, the detection of HSIL/CIN2+ lesions and the proper management of these patients are necessary so that they do not develop into invasive diseases [17]. Until now, both international guidelines and national protocols use colposcopy with acetic acid and subsequent histological examination of the suspicious tissue as the gold standard of diagnosis. However, the international scientific community faces the challenge of creating new diagnostic tools, which will have the ability to detect cervical lesions during colposcopy with a high positive predictive value. Thus, patients will benefit by diagnosing lesions before they become visible with acetic acid and without waiting for histological results [28,29].

Another thing that should seriously be taken under consideration, is the subjective nature of the diagnosis made through colposcopy. It is known that the decision whether to send a nonhistological tissue examination is a decision of the doctor performing the colposcopy. Several studies have found no changes in overall colposcopy performance between more and less experienced colposcopists, but substantial variations in PPV and sensitivity [30,31,32]. Junior colposcopists exhibited higher sensitivity but a lower PPV than senior colposcopists. This translates to a greater number of biopsies retrieved clinically. This, in turn, results in increased sensitivity at the expense of decreased PPV [20]. Wei B. et al. [33], on the other hand, found that senior colposcopists had a much greater incidence of detecting HGSIL lesions in terms of accuracy, specificity, and sensitivity.

The use of Zedscan gives quite promising results that allow us to look forward to its complementary use in the future as an adjunct to classic colposcopy. Data from the literature are limited to five published studies, as the use of this specific tool began in 2017 [17,18,34,35,36]. Three of these are from a single location with a small number of patients, and they investigate whether using ZedScan in conjunction with colposcopy improves sensitivity in finding high-grade lesions at the expense of a slight rise in the number of biopsies [17,36,37]. One study specifically addressed the sensitivity of Zedscan in the detection of lesions associated with HPV infection, which did not show a statistically significant sensitivity of the method for these lesions. Finally, Tidy et al. in 2022 completed a large multicenter study of 5257 participants, confirming that simultaneous colposcopy and ZEDscan increase the detection of HSIL and reduce the number of biopsies performed [18].

It is evident from the literature that there are similar studies to the one presented in this article. From the initial design, the intention was to prepare a study of daily clinical practice that incorporates the use of a colposcopy aid for greater accuracy in obtaining biopsies and diagnosing areas with high-grade lesions. For this reason, the study incorporated characteristics of studies with a similar purpose and in other characteristics shows deviations from the literature. In terms of common features, a sample of approximately 100 women was selected, and all underwent EIS examination and colposcopy, and biopsies were sent for histological confirmation. However, our differentiation from existing studies is that we initially selected the entire sample to consist of women referred for examination due to LGSIL. This choice was made because from a clinical point of view, if the diagnosis of a high-grade lesion is missed in this group of patients, the outcome may be bad. Thus, increasing the sensitivity of colposcopy with the use of EIS has great clinical benefit. In addition, the study was chosen to comment on the demographic profile of participating patients and risk factors for cervical cancer.

Colposcopy alone had a specificity rate of 92% and a sensitivity rate of 85.5% for detecting high-grade lesions in this investigation, which is slightly greater than what is described in the literature [27,28,29]. Moreover, the negative prognostic value (NPV) was 67.6% while the positive prognostic value (PPV) was 97% for the prediction of biopsy results. On the other hand, ZedScan exhibited a sensitivity rate of 89.5% and a specificity rate of 84%, while PPV was 94.4% and NPV 72.4% for the prediction of biopsy results. Furthermore, the overestimation rate was 4% for colposcopy and 12.9% for ZED scan, while the underestimation rate was 21.8% for colposcopy and 7.9% for ZedScan. Last, the proportion of false positive results was 16% for ZedScan and 8% for colposcopy. Interestingly, ZedScan exhibited similarly high rates of sensitivity and specificity, while colposcopy exhibited lower sensitivity and higher specificity, data with no statistical importance (*p* > 0.05). In conclusion, in agreement with the literature, we conclude that ZedScan sensitivity and NPV are greater than simple colposcopy when performed by a junior colposcopist.

After analyzing the data from our sample, no statistically significant relationship emerged with any of the literature-known risk factors for causing uterine cancer, apart from the number of sexual partners. More specifically, the number of women who mentioned more than three partners during their lifetime had a clear correlation with the appearance of the disease. We also discovered that the relationship between sexual partners, invasive cervical carcinoma, and nonmalignant cervical disease is nonlinear. The risk for both malignant and nonmalignant disease is a bit higher but relatively stable for women with four to seven sexual partners [38].

In closing, we would like to summarize that colposcopy is a technique of daily practice for the diagnosis of HGSIL. It is a subjective method, dependent on the experience of the operator and many times unnecessary biopsies are performed to confirm the final diagnosis. According to our conclusions, but also in agreement with the international literature, the diagnostic tool ZedScan has a higher sensitivity and NPV for HSIL detection. These observations also extend to junior colposcopists. It is, therefore, clear that more clinical studies are needed to confirm its reinforcing role in early colposcopic diagnosis and without histological confirmation to integrate it into daily clinical practice. Our study presents similarities to other cohort studies of EIS. On the one hand, the repetition of a clinical trial ensures result validation, fostering confidence in the findings, and on the other hand, the application across different and diverse populations confirms the applicability of the Zedscan in everyday life clinical routine. It is worth emphasizing that the present study consists of a limited sample and although the results appear to be in line with the literature, an extended survey would allow more precise estimates of the confidence intervals of the results. Therefore, we consider that the study has to contribute to scientific knowledge about Zedscan, but more studies are needed to draw safe conclusions.

## 5. Conclusions

In conclusion, this study highlights the potential of ZedScan as a promising adjunctive tool in the early diagnosis of high-grade cervical intraepithelial lesions (HSIL). Our findings underscore the superior sensitivity and negative predictive value of ZedScan over traditional colposcopy, suggesting its valuable role in enhancing diagnostic accuracy, particularly in cases handled by less experienced colposcopists. These results advocate for the integration of ZedScan into routine clinical practice, potentially reducing the need for unnecessary biopsies and improving patient care. However, further extensive clinical investigations are warranted to solidify the clinical utility and establish standardized protocols for the incorporation of ZedScan in regular colposcopic examinations. Its potential to augment the early detection and management of cervical lesions without histological confirmation presents a significant opportunity to enhance cervical cancer screening programs and reduce the burden of disease.

## 6. Ethical Approval

The study was conducted according to the guidelines of the Declaration of Helsinki, and approved by the Institutional Review Board of the University of Athens (protocol code 933/14-11-2017 and date of approval 14 November 2017).

## Figures and Tables

**Table 1:** Demographic characteristics of the study sample.

Demographic Characteristics	
**Age (years)**	
20–35	26
36–50	58
51–64	17
**Race**	
Caucasian	96
Other	5
**Number of sexual partners**	
≤3	58
>3	43
**Systematic condom use**	
yes	79
no	22
BMI	
<25	62
>25	39
**Smoking**	
Yes	62
No	39
**Comorbidities**	
Systematic Lupus Erythematosus	9
Diabetes Melitus	21
Ulcerative Colitis	7
Hypertension	11
**HPV vaccination**	
Yes	14
No	87

**Table 2:** ZedScan, colposcopy and biopsy results (N = 101).

	N	%
ZED SCAN		
Normal	29	28.7
LGSIL	17	16.8
HGSIL	55	54.5
Colposcopy		
Normal	34	33.7
LGSIL	31	30.7
HGSIL	36	35.6
Biopsy		
Normal	25	24.8
CIN 1	31	30.7
CIN 2-CIN 3	45	44.6

**Table 3:** ZED SCAN and colposcopy outcomes in association with biopsy results.

	Biopsy		Results			
	Normal		CIN 1		CIN 2-CIN 3	
	N	%	N	%	N	%
**ZED SCAN**						
Normal	21	84.0	6	19.4	2	4.4
LGSIL	1	4.0	16	51.6	0	0.0
HGSIL	3	12.0	9	29.0	43	95.6
**Colposcopy**						
Normal	23	92.0	11	35.5	0	0.0
LGSIL	2	8.0	18	58.1	11	24.4
HGSIL	0	0.0	2	6.5	34	75.6

**Table 4:** ZED SCAN in association with colposcopy outcome.

	Colposcopy					
	Normal		LGSIL		HGSIL	
	N	%	N	%	N	%
ZED SCAN						
Normal	23	67.6	4	12.9	2	5.6
LGSIL	8	23.5	9	29	0	0
HGSIL	3	8.8	18	58.1	34	94.4

**Table 5:** Sensitivity, Specificity, PPV and NPV for ZED SCAN and Colposcopy.

	Sensitivity %	Specificity %	PPV * %	NPV * %	AUC (95% CI)	*p*
ZED SCAN	89.5	84.0	94.4	72.4	0.87 (0.78–0.96)	<0.001
Colposcopy	85.5	92.0	97.0	67.6	0.89 (0.81–0.97)	<0.001

* PPV = Positive Predictive Value; NPV = Negative Predictive Value.

## Data Availability

Not applicable.

## Abbreviations

ASC-H: atypical squamous cells of undetermined significance without excluding high-grade squamous intraepithelial lesions; ASCUS: Atypical Squamous Cells of Undetermined Significance; AUC: Area Under the Curve; CIN: Cervical Intraepithelial Neoplasia; CIN2+: Cervical Intraepithelial Neoplasia Grades 2+; EIS: Electrical Impedance Spectroscopy; HG-CIN: High-grade Cervical Intraepithelial Neoplasia; HPV: human papillomavirus; HSIL: High grade squamous intraepithelial lesion; HGSIL: High grade squamous intraepithelial lesion; NILM: Negative for Intraepithelial Lesion or Malignancy; LBC: Liquid-Based Cytology; LSIL: Low-grade Squamous Intraepithelial Lesion; LGSIL: Low-grade Squamous Intraepithelial Lesion; NPV: Negative Predictive Value; SCC: Squamous Cervical Carcinomas; SD: Standard Deviations; ROC: Receiver Operating Characteristic; SCJ: squamocolumnar junction; TZ: Transformation Zone.
